# Guillain-Barré Syndrome Surveillance during National Influenza Vaccination Campaign, New York, USA, 2009

**DOI:** 10.3201/eid1912.130643

**Published:** 2013-12

**Authors:** Gregory P. Giambrone, Shelley M. Zansky, Millicent Eidson, Pamela G. Duncan, Louise-Anne McNutt, Guthrie S. Birkhead

**Affiliations:** New York State Department of Health, Albany, New York, USA (G.P. Giambrone, S.M. Zansky, M. Eidson, P.G. Duncan, G.S. Birkhead);; University at Albany School of Public Health, Rensselaer, New York, USA (L.-A. McNutt, G.S. Birkhead)

**Keywords:** Guillain-Barré syndrome, GBS, pandemic H1N1 2009 influenza virus, influenza A(H1N1) 2009 monovalent vaccine, vaccination campaign, viruses, New York

## Abstract

The New York State Department of Health (NYSDOH) collected information about hospitalized patients with Guillain-Barré syndrome (GBS) during October 2009–May 2010, statewide (excluding New York City), to examine a possible relationship with influenza A(H1N1)pdm09 vaccination. NYSDOH established a Clinical Network of neurologists and 150 hospital neurology units. Hospital discharge data from the Statewide Planning and Research Cooperative System (SPARCS) were used to evaluate completeness of reporting from the Clinical Network. A total of 140 confirmed or probable GBS cases were identified: 81 (58%) from both systems, 10 (7%) from Clinical Network only, and 49 (35%) from SPARCS-only. Capture–recapture methods estimated that 6 cases might have been missed by both systems. Clinical Network median reporting time was 12 days versus 131 days for SPARCS. In public health emergencies in New York State, a Clinical Network may provide timely data, but in our study such data were less complete than traditional hospital discharge data.

In the fall of 1976, the outbreak of a swine-origin influenza virus prompted a mass vaccination campaign in the United States. Although an influenza epidemic did not occur, epidemiologic investigations demonstrated a small but significant risk for Guillain-Barré syndrome (GBS) among adult vaccine recipients within 6 weeks after vaccination (*1*–*4*). Some studies found that a relatively small risk extended slightly beyond the 6 weeks after vaccination (*1*,*2*). The estimated attributable risk for GBS after swine influenza vaccination was slightly less than 1 case per 100,000 persons vaccinated (*1*,*3*,*4*). Because of this association, GBS surveillance was established for the 3 subsequent influenza seasons; however, no increased risk for GBS was identified after influenza vaccination (*5*,*6*). The underlying reason for the association with the 1976 vaccination remains unknown.

In April 2009, influenza A(H1N1)pdm09 virus was first identified (*7*–*9*). Its emergence and rapid global spread prompted swift development of a new vaccine. The previous association of GBS with the 1976 vaccine raised concerns about the potential for a similar association with the new A(H1N1)pdm09 monovalent vaccines.

In June 2009, the Centers for Disease Control and Prevention (CDC) engaged the 10 CDC-funded Emerging Infection Program (EIP) sites (*10*,*11*), including New York State (NYS), to rapidly collect and report information about hospitalized persons with GBS during October 1, 2009–May 31, 2010, to examine a possible relationship with A(H1N1)pdm09 vaccines. Some participating sites had the capability to collect hospital discharge data in real time and used this method as a primary reporting source. However, NYS has inherently long delays in hospital discharge data reporting, so to conduct real-time surveillance, NYS established a network of practicing neurologists as primary reporters. Hospital discharge data were used to supplement and retrospectively evaluate the completeness of the active physician-based reporting system.

Results of the overall national EIP GBS surveillance system during the A(H1N1)pdm09 vaccination campaign, which includes NYS data from hospital discharge data and the physician-based reporting system, have been described (*10*,*11*). Because of the rarity of GBS and the small excess risk identified by multistate efforts associated with A(H1N1)pdm09 vaccines (*10*,*11*), the NYS Department of Health’s (NYSDOH) EIP did not attempt to study the association between vaccination and GBS. Presented here is a comprehensive evaluation of the NYSDOH EIP’s use of a neurologist-based reporting surveillance system. Capture–recapture was used to compare hospital discharge data with neurologist reports to evaluate the completeness of the overall NYSDOH surveillance system.

## Materials and Methods

### Data Sources

NYSDOH conducted surveillance for hospitalized persons with GBS who were admitted during October 1, 2009–May 31, 2010, among NYS residents, excluding New York City. The total population under surveillance was ≈11.1 million persons.

#### Neurologist-based GBS Surveillance

Under the authority of NYS Public Health Law 206(1)(j), the NYSDOH Commissioner approved a time-limited request for physician reporting of GBS, not usually a notifiable condition. This request enabled NYSDOH to establish real-time surveillance by asking neurologists to report all suspected GBS cases. The NYSDOH Institutional Review Board approved the surveillance protocols.

##### Neurologists

Licensed neurologists practicing in NYS were identified in 2 ways: from a list from the NYSDOH Physician Profile System, in which all licensed physicians are required to maintain updated information (e.g., their specialty, practice location), and from a list provided by the American Academy of Neurology of NYS members. These lists were combined to create a singular deduplicated dataset with current address information for >2,600 neurologists.

##### Hospital-based Neurology Units

To focus surveillance efforts, study staff analyzed hospital discharge data to identify high-volume hospitals (i.e., hospitals that treated >5 GBS cases during 2003–2008) and lower-volume hospitals (i.e., hospitals that treated <5 GBS cases during 2003–2008). Of 183 NYS hospitals, 101 high-volume hospitals treated 95% of GBS cases diagnosed during 2003–2008. Study staff contacted the 101 high-volume hospitals to identify whether a neurology inpatient clinical unit was present or whether a private practice provided the hospital’s neurology inpatient services. These inquiries produced 150 neurology clinical units/neurology practices recruited as active reporters. A second group of passive reporters comprised the remaining neurologists.

Therefore, 2 groups were created: 1) 150 active reporting sites based in hospital clinical neurology units or neurology practices and 2) 2,494 passive reporting neurologists identified through the deduplicated physician list. We refer to this combined group of active and passive reporters as the Clinical Network. Active reporters were mailed an information packet in mid-October 2009 that included a letter emphasizing the importance of reporting and the authority under which surveillance was conducted and a standardized case report form. Contact information was requested for a person at the practice or hospital who could serve as liaison. To facilitate reporting of suspected GBS, the liaison at the 150 active reporting sites received a biweekly email or phone call in accordance with the facility’s preference. Some hospitals identified infection preventionists as the primary reporters. The 2,494 neurologists in the passive reporting group received the initial informational packet but no biweekly follow-up. In early March 2010, both groups received a second letter emphasizing the importance of continued reporting and providing a summary of preliminary results.

#### Hospital Discharge Data GBS Surveillance

New York’s Statewide Planning and Research Cooperative System (SPARCS) collects administrative data on all hospital discharges in NYS. Reporting facilities are required to submit 95% of hospital discharge data within 60 days after the discharge month and 100% within 180 days after the facility’s fiscal year ends. Because these delays render SPARCS ineffective as a primary real-time reporting system, we used SPARCS as a secondary system to retrospectively evaluate completeness of reporting from the Clinical Network. Staff reviewed SPARCS data monthly beginning January 1, 2010, for a primary or secondary GBS discharge diagnosis (International Classification of Diseases, Ninth Revision, Clinical Modification, code 357.0 [acute infective polyneuritis]) in NYS residents who were admitted during the study period. Because of SPARCS reporting delays, review continued through November 2010 to capture at least 95% of all admissions during the study period that had a GBS discharge code.

### GBS Case Definition

Case status was determined by using the Brighton Collaboration Case Criteria for GBS, which incorporates 7 clinical and 2 diagnostic study criteria in hospitalized patients (*12*). Before staff carefully reviewed medical records, all patients reported as having GBS and/or having a primary or secondary GBS diagnosis code in SPARCS during the study period were considered to have suspected cases.

After medical record review, cases were assigned to 1 of 3 case definitions: confirmed, probable, and noncase. Confirmed case-patients met all clinical criteria and at least 1 diagnostic study criteria; probable cases met all clinical criteria but did not meet diagnostic study criteria; and noncases did not fulfill clinical criteria or an alternative diagnosis was provided. Methods used for case classification have been described (*10*,*11*).

### Follow-up of Reported Cases

NYSDOH staff followed up on all reported GBS cases, regardless of reporting source. For patients transferred between facilities, medical records were reviewed at the hospital where most of the diagnostic work-up and treatment was provided. Medical record reviews were conducted by using a standardized CDC Medical Record Review Form to assess case status, patient vaccination history (for both A[H1N1]pdm09 and seasonal influenza), and additional variables of interest in accordance with CDC protocol (*10*). The study coordinator discussed any unclear or missing information after medical record review with the case-patients’ consulting neurologists and CDC. To ensure accurate and timely results, NYSDOH contracted with 11 public health and hospital-based nurses throughout NYS trained to conduct onsite medical record reviews. Staff conducted voluntary patient interviews with all confirmed and probable case-patients or their family members by using a standardized interview form. Vaccine histories were collected and verified by using 4 sources: medical record review, patient interview, information from primary care providers, and the NYS Immunization Information System.

### Data Analysis

We comprehensively evaluated data obtained through the Clinical Network and SPARCS. Using medical record reviews as a standard, we calculated positive predictive values (PPVs) and compared them for the Clinical Network and SPARCS. Cohen’s κ coefficient was used to assess overall agreement between the 2 systems and data reliability.

Capture–recapture methods have been applied to epidemiologic data (*13*–*18*). Therefore, to evaluate the completeness of the overall GBS surveillance system, we used Chapman capture–recapture methods using 2 data sources (*19*). By matching case-patients identified through the Clinical Network and SPARCS on sex, birth, and admission and discharge dates, we calculated an estimate of the total number of GBS cases and its 95% CI (*19*,*20*). The total number of GBS cases was estimated by n = [(*b+*1)(*c+*1)/(*a+*1)] *− *1, where *b* and *c* are the numbers of persons in the first and second capture, respectively, and *a* is the number identified in both captures (*19*).

We used standard definitions to compare timeliness of reporting and timeliness to review of the Clinical Network and SPARCS. Time to report was defined as the difference, in days, between patient’s hospital admission date and date a report was received by NYSDOH. Median time to report was compared between the 2 reporting systems. Time to review was defined as the difference, in days, between the date a report was received by NYSDOH and date of the medical record review. Median time to review was compared between the 2 reporting systems.

We evaluated reporting completeness by comparing Clinical Network cases with cases identified through SPARCS-only. To assess for biases in Clinical Network case reporting, SPARCS-only cases were reviewed to identify reporting differences by admission date, average age, sex, antecedent events, active/passive reporters, and A(H1N1)pdm09 vaccination status. Variables were compared by reporting source (Clinical Network vs. SPARCS) using Fisher exact test with p<0.05 considered statistically significant.

Data were stored in Microsoft Access 2007 (Microsoft, Redmond, WA, USA). Data were cleaned and analyzed by using Microsoft Excel 2007 (Microsoft) and SAS software, version 9.1 (SAS Institute, Cary, NC, USA).

## Results

### Case Reports

NYSDOH received 576 suspected GBS cases from the combined Clinical Network and SPARCS surveillance system among residents with hospital admission dates during October 1, 2009–May 31, 2010 (Figure). All 240 reported patients who met study eligibility requirements were reviewed and assigned a case status, 140 were classified as having confirmed/probable GBS (Figure). When the Clinical Network data were compared with SPARCS data, 81 cases were identified in both systems, 10 cases were identified by the Clinical Network only, and 49 cases were identified by SPARCS-only (Figure). SPARCS detected 130 of the confirmed cases (sensitivity 92.9%); the Clinical Network detected 91 confirmed cases (sensitivity 65.0%). PPV was higher for the Clinical Network (82%) than for SPARCS (59%). Cohen’s κ coefficient was 0.52, indicating moderate agreement between the 2 reporting sources (*21*).

**Figure:**
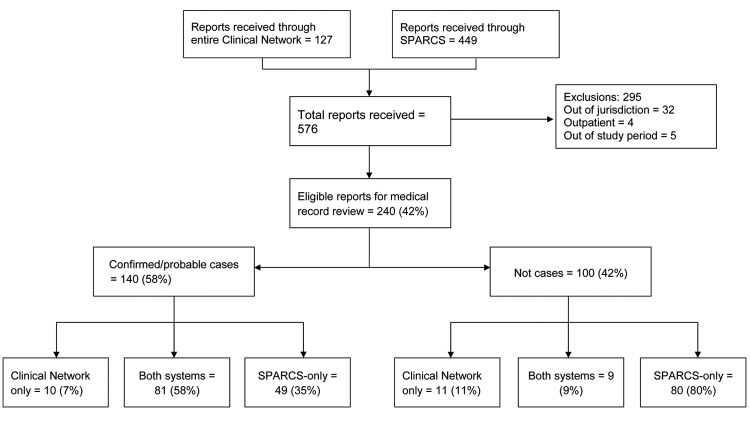
Surveillance for Guillain-Barré syndrome during the A(H1N1)pdm09 National Influenza Vaccination Campaign, New York State, USA, October 1, 2009–may 31, 2010. SPARCS, Statewide Planning and Research Cooperative System.

### Capture–Recapture Analysis

Capture–recapture analysis indicated that the entire NYSDOH surveillance system missed only 6 cases, yielding 146 (95% CI 140–152) GBS cases. Thus, NYSDOH surveillance identified 96% of the estimated cases.

### Timeliness of Case Reporting and Medical Record Review

For SPARCS, median time to report was 131 days after hospital admission (Table 1). In contrast, the Clinical Network had a median time to report of 12 days after hospital admission. Medical records for reports identified through the Clinical Network were reviewed within a median of 7 days, compared with 14 days for SPARCS reports.

**Table 1:** Timeliness of reporting data to NYSDOH Guillain-Barré syndrome surveillance system, 2009–2010*

Reporting source, time, d	Mean	Median	Range
Clinical Network†			
To report‡	18	12	0–127
To review§	9	7	0–42
SPARCS¶			
To report	130	131	58–196
To review	28	14	4–184

*NYSDOH, New York State Department of Health; SPARCS, Statewide Planning and Research Cooperative System. †Network of >2,494 reporting clinical neurologists. ‡Time lapse between patient’s hospital admission date and NYSDOH receipt of report. §Time between date NYSDOH received report and medical record review. ¶SPARCS collects administrative data on all hospital discharges in the state.

### Analysis of Hospital Discharge Data as a Primary Reporting System

When analyzing SPARCS as the sole reporting source, 219 GBS patient reports were identified, of which 130 (59%) met confirmed/probable case definition (Table 2) and 89 (41%) were determined upon review to be false positive. However, when data were stratified by primary and secondary diagnosis, using only a primary GBS diagnosis code, 116 (89%) of the total 130 cases were identified, and PPV increased from 59% to 78% (Table 2).

**Table 2:** Use of primary and secondary diagnosis codes for GBS identified by SPARCS, New York State Department of Health GBS surveillance system, 2009–2010*

Diagnosis	Total cases reported	Confirmed/probable cases, no. (%)
Primary	149	116 (78)
Secondary	70	14 (20)
Total	219	130 (59)

*GBS, Guillain-Barré syndrome; SPARCS, Statewide Planning and Research Cooperative System. SPARCS collects administrative data on all hospital discharges in state facilities.

### Assessment of Bias in Clinical Network Reporting

The 49 confirmed/probable cases missed by the Clinical Network were reviewed further to identify possible biases in reporting. For 17 (35%) cases, admission date was either early or late in the surveillance period, with 8 cases missed in October 2009 and 9 missed in May 2010. No differences were found between case-patients in SPARCS-only and in the Clinical Network on the basis of sex (male 59% vs. 52%, respectively), age (mean 54.5 years vs. 53.3 years), or antecedent event 1–6 weeks before GBS symptom onset (65% vs. 56%). Among 91 Clinical Network–reported cases, 82 (90%) were received from active reporters and 9 (10%) from passive reporters. Among 49 SPARCS-only cases missed by the Clinical Network, 42 (86%) patients were under the care of active reporters and 7 (14%) were under the care of passive reporters.

### Exposure to A(H1N1)pdm09 Vaccines

Nineteen (14%) of 140 patients with confirmed/probable GBS received A(H1N1)pdm09 vaccine (Table 3); GBS developed in 8 (42%) of persons within 1–6 weeks after vaccination and in 11 (58%) >6 weeks after vaccination. Six (75%) of the 8 patients with confirmed/probable GBS that developed within 1–6 weeks after vaccination were identified by the Clinical Network, and 2 (25%) were identified by SPARCS-only. Of the 91 confirmed/probable Clinical Network–reported cases, 14% received A(H1N1)pdm09 vaccine before GBS diagnosis. This proportion is similar to that found by the total surveillance system (14%) and that found if SPARCS was the stand-alone system (15%) (Table 3). Although not statistically significant, a difference was noted between the 2 surveillance systems related to recording vaccination history in the medical record. Of 19 confirmed/probable GBS case-patients who received A(H1N1)pdm09 vaccine, 9 (69%) of 13 Clinical Network–reported cases had vaccination status noted in the medical record, compared with 2 (33%) of 6 SPARCS–identified cases.

**Table 3:** Vaccination status of patients with confirmed or probable GBS, New York State Department of Health GBS surveillance system, 2009–2010*-

Reporting source	Total confirmed/probable GBS case-patientss	A(H1N1)pdm09 monovalent vaccine status	
		Received, no. (%)	Did not receive, no. (%)
Clinical Network† and SPARCS‡	140	19 (14)	121 (86)
SPARCS	130	19 (15)	111 (85)
Clinical Network	91	13 (14)	78 (86)
SPARCS-only¶	49	6 (12)	43 (88)

*GBS, Guillain-Barré syndrome; SPARCS, Statewide Planning and Research Cooperative System. †Network of >2,494 reporting clinical neurologists.  ‡SPARCS refers to all suspected cases identified through hospital discharge data (some of these cases might have been identified by the Clinical Network as well). ¶Cases missed by the Clinical Network and identified only through SPARCS.

## Discussion

National active GBS surveillance was implemented at EIP sites to assess whether a statistically significant association existed between GBS and A(H1N1)pdm09 vaccines. NYSDOH contributed a quarter of the total population under surveillance to national EIP efforts, identifying suspected cases by using physician-based reporting and SPARCS. NYSDOH’s use of a Clinical Network as primary reporters and SPARCS as a supplementary source was an effective and complete method of GBS surveillance, identifying ≈96% of all GBS cases in the study population. The Clinical Network had a high PPV (82%) and its data were timely but lacked completeness, identifying 65% of total cases. However, the Clinical Network and the overall surveillance system had an equal proportion (14%) of case-patients vaccinated for A(H1N1)pdm09 virus, suggesting that using only the Clinical Network would not have biased results related to vaccination. During this emergency situation, timeliness and a high PPV for the primary source of reporting (i.e., the Clinical Network) were vital to ensure timely review and transmission of data to CDC for further analysis of vaccine safety. If the Clinical Network had a low PPV, as was seen in SPARCS (59%), our capacity to rapidly review medical records would have been diminished. An influx of false-positive reports would have delayed the time to review and delayed transmission of complete data to CDC.

The rapid time to report and the quantity of reports received from the Clinical Network demonstrated a strong collaboration among the clinical neurology community, infection preventionists, and public health authorities. The Clinical Network provided timely reporting with a median time to report of 12 days. An evaluation of US public health infectious disease reporting systems that used public health and biomedical literature found a median of 12 days (1–54 days) for meningococcal disease from diagnosis to initiation of investigation by the state public health agency and a median of 21 days (2–41 days) for *Escherichia coli* O157:H7 (*22*) infection. Therefore, the Clinical Network’s and NYSDOH’s time to report and investigate cases (median 19 days [12 days to report, 7 days to review]) was comparable to that for infectious disease surveillance systems. This finding suggests that during a potential public health crisis, neurologists, who may be unaccustomed to reporting to public health authorities, may be timely and competent reporters if the reasons to report are compelling and clear and the Commissioner of Health has officially requested reporting.

Adequate staffing is necessary for successful surveillance, and contracting public health/hospital-based nursing staff enabled timely review of medical records. Staff reviewed cases identified through the Clinical Network in a median of 7 days versus 14 days for SPARCS. SPARCS cases had a longer time to record review because many of these patients had been discharged and medical records departments required additional time to locate records. Many case-patients reported through the Clinical Network were hospitalized at the time of review, enabling easier access to medical records and thus a quicker review process.

If SPARCS had been the sole reporting source, using a primary diagnosis code of 357.0 would have identified 83% of total cases, including all cases in vaccinated persons. A routine GBS surveillance system based solely on hospital discharge data would be substantially less resource dependent (because of its ability to batch medical record reviews and its need for fewer staff) and have high sensitivity. The Clinical Network required a full-time staff member, 2 research assistants to maintain data and conduct biweekly follow-up calls to the neurology practices, and 11 study nurses to review medical records. Time invested by the neurology practices, infection preventionists, and medical records departments also must be considered in the overall cost of establishing and maintaining such a robust surveillance system. This evaluation has shown that for future surveillance efforts involving GBS, using a primary diagnosis code of 357.0, requires fewer resources than establishing a Clinical Network, has a high PPV, and can identify a high proportion of all GBS cases. However, in NYS, these costs were necessary because of the lack of timeliness of SPARCS. For this surveillance system, even if SPARCS were timely, it would still carry a substantial cost because of the intensive follow-up required. Receipt of reports took a median of 11 times longer from diagnosis date through SPARCS and double the time to review than through the Clinical Network. Reporting timeliness is a key surveillance system metric, and its importance is specific to the health-related event under surveillance (*23*). For GBS surveillance related to vaccination, the long delay in SPARCS reporting was unacceptable. The mass vaccination campaign necessitated rapid collection and analysis of data to determine vaccine safety and reassure providers and the public.

The Clinical Network may have failed to identify 49 cases found through SPARCS-only for several reasons. Because of logistical issues, information packets notifying the Clinical Network of the surveillance system were not sent until mid-October 2009, and NYSDOH staff did not receive a contact person for most of active reporters for biweekly follow-up until early November 2009. Many neurologists did not review their records retrospectively to October 1, 2009, and instead reported prospectively from mid-October 2009, causing underreporting in October. Reporter fatigue was noted late in the surveillance period, with only 4 confirmed/probable Clinical Network reported cases in May 2010. Supporting the suspicion that underreporting was largely a logistical issue, no differences based on demographic or vaccination status were identified between cases identified by the Clinical Network and SPARCS-only cases. Although a statistically significant difference was not identified, some reporters expressed confusion about whether to report GBS in nonvaccinated patients throughout the surveillance period.

Our conclusions are subject to 2 possible limitations. First, some GBS cases might have been misidentified; however, this circumstance was minimized by use of standardized case definitions and standardized training of the surveillance officer and contracted nursing staff. In addition, all reported GBS cases received equal follow-up regardless of reporting source, including discussing the case with the patient’s consulting neurologist when information was unclear or missing. Second, specificity values could not be calculated because there was no standard for comparison; therefore, Cohen’s κ coefficient was used to assess the reliability of the data. Sensitivities of 65% and 93% were found for the Clinical Network and SPARCS, respectively, but these sensitivities may be overestimated because of the possible cases missed by both systems. However, capture–recapture analysis suggests that few cases were missed.

When a credible public health emergency arises, physicians unaccustomed to reporting noninfectious diseases might be asked for assistance to protect public health and safety. For emergency GBS reporting, the Clinical Network reported quickly and well with a high PPV, and record reviews were conducted rapidly by using contracted nursing staff. These efforts led to the prompt transmission of data to CDC for the timely analysis of vaccine safety. The Clinical Network did not achieve complete case ascertainment in comparison with hospital discharge data, but no systematic bias with regard to A(H1N1)pdm09 vaccination status was evident, so data were judged valid for inclusion in the national multistate study of the risk for GBS in persons receiving A(H1N1)pdm09 vaccine. However, because of the resources needed to develop and maintain this system, it is not recommended for routine surveillance or research studies. Hospital discharge data can be used for nonemergent situations, routine surveillance, and research, but users should be aware of the built-in eporting delays.
